# Lessons from SARS: A retrospective study of outpatient care during an infectious disease outbreak

**DOI:** 10.1186/1471-2431-10-51

**Published:** 2010-07-20

**Authors:** Nehad Nasef, Karel O'Brien, Lesley Wylie, Sharon Unger

**Affiliations:** 1 Paediatrics, Mount Sinai Hospital, Toronto, Ontario, Canada; 2 Neonatal Neurodevelopmental Follow Up, SickKids, Toronto, Ontario, Canada

## Abstract

**Background:**

During severe acute respiratory syndrome (SARS) outbreak in Toronto, outpatient clinics at SickKids Hospital were closed to prevent further disease transmission. In response, a decision was made by the neonatal neuro-developmental follow up (NNFU) clinic staff to select patients with scheduled appointments to have a mail/telephone assessment using Ages and Stages Questionnaire (ASQ) or to postpone/skip their visit. The objective of this study was to compare the developmental assessment and its outcome in two groups of NNFU clinic patients, SARS versus non-SARS, over three standard clinic appointments.

**Methods:**

We compared the diagnostic accuracy (identification of developmental delay), and patient management (referral for therapy or communication of a new diagnosis) of the strategies used during SARS, April/May 2003, to the standard assessment methods used for patients seen in April/May 2005 (non-SARS). In all cases data were obtained for 3 patient visits: before, during and after these 2 months and were compared using descriptive statistics.

**Results:**

There were 95 patients in the SARS group and 99 non-SARS patients. The gestational age, sex, entry diagnosis and age at the clinic visit was not different between the groups. The NNFU clinic staff mailed ASQ to 27 families during SARS, 17 (63%) were returned, and 8 of the 17 were then contacted by telephone. Criteria used to identify infants at risk selected for either mailed ASQ or phone interviews were not clearly defined in the patients' charts. There was a significant under identification of developmental delay during SARS (18% versus 45%). Of those who responded to the mailed questionnaire, referrals for therapy rates were similar to non-SARS group. The lost to follow up rate was 24% for the SARS group compared with 7% for non-SARS. There was no difference in the overall rate of developmental delay in the two groups as identified at the 'after' visit.

**Conclusions:**

Poor advanced planning led to a haphazard assessment of patients during this infectious disease outbreak. Future pandemic plans should consider planning for outpatient care as well as in hospital management of patients.

## Background

Care in neonatal intensive care units has progressed over the past years resulting in higher number of surviving preterm extreme low birth weight infants as well as high risk full term infants [1-4]. These infants are at an increased risk for developmental delay that can arise at any time along their developmental trajectory [5]. Neonatal neuro-developmental follow up (NNFU) clinics were designed for effective monitoring and assessment of these infants at regular time intervals. Regular attendance at scheduled appointments by children with their parents or care givers and sensitive feedback of the assessment to the family are important factors in early prediction of neuro-developmental delay in high risk infants [6].

In spite of efforts made to accommodate care givers there is still a significant number of missed appointments and loss to follow up at most neonatal follow up clinics due to factors related to the parents or care givers [7]. Thus, alternative approaches for obtaining outcome data, including home visits by trained lay interviewers, telephone interviews, in-person interviews in a clinic, and mailed questionnaires have been evaluated [8].

Infectious diseases such as SARS (Severe Acute Respiratory Syndrome) are an important cause of changing routine health practice due to closure of non emergency health services to control disease spread [9-11]. SARS spread to the Greater Toronto Area on February 23rd, 2003 following which there were two phases of the outbreak. The first outbreak was believed to be over after passage of two incubation periods in early May. However, on May 20, 2003 new cases of respiratory illness occurred in a rehabilitation facility and a second outbreak was identified [12,13]. A provincial emergency was declared throughout the two outbreaks from the end of March to the middle of June. As part of government imposed containment directives, all elective outpatient hospital clinics were closed between March 2003 and June 2003 [14,15]. Hospital staff was expected to continue to come to work for regularly scheduled shifts.

High risk preterm and full term infants are regularly scheduled for neuro-developmental assessment in our NNFU clinic to provide diagnosis and referral for therapy. As a consequence of SARS all outpatient clinics were closed. Concerns about missed appointments in the NNFU clinics and the impact this may have on patients and their families led the NNFU team at The Hospital for Sick Children and Mount Sinai Hospital in Toronto to use an alternative method of assessment. A decision was made to use a mailed questionnaire and a telephone interview rather than the standard face to face patient assessment. The Ages and Stages questionnaire (ASQ) was mailed to parents for them to complete.

The objective of this retrospective study was to compare the developmental assessment and its' outcome in two groups of NNFU clinic patients, SARS versus non-SARS over an assessment trajectory of 3 booked clinic appointments (labeled before, during and after according to the time of clinic closure during SARS). The outcome measures explored included diagnostic accuracy, appropriate referral and return to follow up rate.

## Methods

This was a retrospective study comparing the management of 2 groups of patients in the NNFU program at The Hospital for Sick Children and Mount Sinai Hospital in Toronto, Canada. Charts for all infants enrolled in the program during the SARS outbreak (SARS group) were reviewed for the accuracy of developmental assessment for the period before, during and after the SARS containment period. The comparator was patients enrolled in the program two years later who were identified as the non-SARS group. The study was approved by the Research Ethics Boards at Mount Sinai Hospital and The Hospital for Sick Children.

### Study population

Infants were identified as eligible for neuro-developmental follow up in our program if either preterm (≤27 weeks gestation) or having other pre or post natal risk factors for adverse neuro-developmental outcome. SARS group infants were identified as those whose NNFU appointments were planned but subsequently cancelled during the period of April and May 2003 due to SARS. Non-SARS group infants had planned appointments in the corresponding time period of April and May 2005.

### Data Collection

Data were abstracted from the NNFU chart including demographics, reason for referral and results of developmental assessment. The SARS period was defined as April to May 2003 and was labeled as 'during' visit. The visit immediately prior to this was labeled 'before' and visit 3 as 'after'. The non-SARS group assessments were matched for the corresponding time periods in 2005. Data collection included the type of assessment used and identification of a developmental delay in any of: gross motor, fine motor, language and socio-adaptive skills. Other data regarding referral for further therapy or further testing and the delivery of a new diagnosis were also reviewed.

### Developmental assessment

The developmental assessment visits at our NNFU are scheduled at 4, 8, 12 and 20 months corrected age. Neuro-developmental assessments are performed by an inter-disciplinary team of physicians, nurses and therapists (occupational, physical and speech). Hearing, vision, neuromuscular and cognitive development are assessed using standardized tools. During the SARS period (during visit), as a replacement for a usual visit, the ASQ [16], a standardized parent completed survey assessing five domains of development (gross motor, fine motor, problem solving, socio-adaptive and language) was mailed to parents. Upon its' return there was an attempt to make a follow up telephone call to confirm the report. A developmental delay was defined as testing below the cutoff in any domain.

### Data analysis and statistical tests

Data were analyzed to compare the rate of reported developmental delay as well as the rate of referral for further therapy or further testing between SARS group and non-SARS group during each of the three studied visits. The rate of new diagnoses communicated with a family was also compared.

Statistical analysis was performed using Microsoft Access and Excel (Microsoft Corp., Chicago, IL, USA). Student t test was used to compare demographic characteristics, while Chi Square independent test or Fischer's exact test was used to compare categorical developmental data (developmental delay, yes or no). A p < 0.05 was considered statistically significant.

## Results

### Demographics/Patient management

Ninety-five patients (mean gestational age of 32.1 ± 5.1 weeks gestation) had planned but cancelled appointments in NNFU during the SARS period (SARS group) and 99 patients (mean gestational age of 32.7 ± 5.6 weeks gestation) were seen during the corresponding time 2 years later (non-SARS group). There were no significant differences between groups for demographics or reason for NNFU enrollment (Table 1).

**Table 1:** Demographic data

	SARS Group	Non-SARS Group
	n = 95	n = 99
Gestational Age (weeks)	32.1 ± 5.1	32.7 ± 5.6

Birth Weight (grams)	1885 ± 1145	1980 ± 1138

Male Sex	54 (56.8%)	51 (51.5%)

Preterm	58 (61%)	49 (49.5%)

PPHN	13 (13.7%)	14 (14.1%)

HIE	8 (8.4%)	11 (11.1%)

IUGR	5 (5.2%)	8 (8%)

ICH	2 (2.1%)	3 (3%)

Multiple gestation	7 (7.3%)	9 (9.1%)

Neonatal drug withdrawal	2 (2.1%)	5 (5%)

Data are expressed as either mean ± SD or number and percentage

p > 0.05 between all groups

PPHN = persistent pulmonary hypertension; HIE = hypoxic ischemic encephalopathy; IUGR = inutero growth restriction; ICH = intracranial hemorrhage

**Figure 1:**
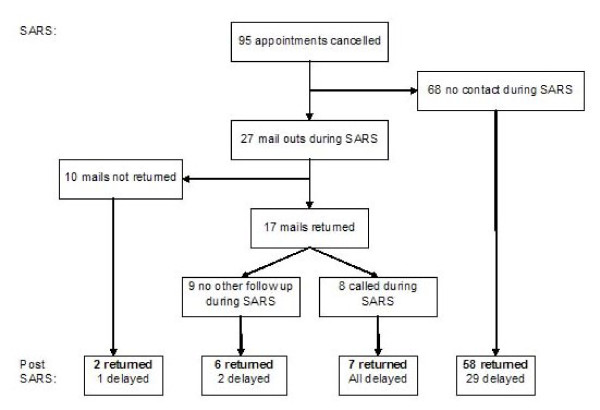
**Study patient assessments**.

The ASQ was mailed to 27 infants' families during the SARS period, representing 28.4% of those with planned appointments. Seventeen families returned the questionnaire (63%) and of those, eight had a telephone interview (Figure 1). Criteria used to identify infants at risk selected for either mailed ASQ or phone interviews were not clearly defined and were left to the discretion of the team members following the infants. Of 30 patients diagnosed with developmental delay at the before visit in the SARS group, 8 were contacted by mail or telephone and 22 were never contacted during the period of clinic closure.

### Identification of Developmental Delay

Using the ASQ information, the follow up team was able to identify 3 children (18%) with developmental delay during SARS, while 14 children (82%) were interpreted as being normal. Although there was an apparent difference in age of those diagnosed with a delay (18.0 ± 3.4 versus 12.9 ± 4.0 months), this did not reach statistical significance. Of the three infants diagnosed with developmental delay by ASQ during SARS, only two were confirmed by face to face assessment on their subsequent visit. Of the 14 infants with a normal ASQ, five were found to be developmentally delayed by face to face assessment at the after visit. When comparing assessments made during SARS (during visit) to non-SARS during the corresponding period 2 years later, we found that the rate of identification of developmental delay was significantly lower during SARS (18% versus 45%, p = 0.03) (table 2). The rate of identified developmental delay was not significantly different between SARS group and non-SARS group in the visits prior to (before visit) or after the study period (after visit) (Table 2).

**Table 2:** Rate of Identification of Developmental Delay and Referral for Therapy

	Developmental delay		Referral for therapy	
	
	SARS	Non-SARS	SARS	Non-SARS
"before" visit	32% (30/95)	32% (32/99)	20% (19/95)	24% (24/99)

"during" visit	18% (3/17)*	45% (45/99)*	12% (2/17)	26% (26/99)

"after" visit	53% (39/73)	46% (43/94)	32% (23/73)	33% (31/94)

Data are expressed as percentage (absolute number)

*p < 0.05

### Rate of referral for therapy/further testing

The rate of referral for therapy or testing was not significantly different between the SARS and non-SARS group over the span of the three visits. The apparent difference in the during visit (12% versus 26%) did not reach statistical significance.

### Rate of new diagnosis

Of the SARS group, the follow up team identified 4 infants with cerebral palsy as well as 2 infants with autism in the visit after SARS. There were no new neurological diagnoses made remotely during the SARS period. None of the newly diagnosed children with cerebral palsy or autism in the after visit had been evaluated during the SARS period. In contrast to the SARS group, the follow up team was able to identify new diagnoses among the non-SARS group throughout the three visits using direct face to face assessment, 4 at the during visit and 1 at the after visit.

### Loss to follow up

Data for the period prior to SARS (before visit) were available for all infants while data for 73 infants were available following SARS (after visit) representing a 24% (22/95) loss to follow up rate over the three visits compared to 5% (5/99) for non-SARS group over the same period.

## Discussion

This study clearly illustrates that in absence of a priori planning, patient care and contact was unorganized and provided haphazardly in a non-urgent care clinic during the time of an infectious disease outbreak. Other reports have focused on inpatient care, where non urgent hospital utilization and hospital transfers were restricted, but this is the first such report of the impact on a hospital based clinic from the SARS time period [17,18].

There were 95 patients with cancelled appointments during the SARS period and the NNFU team made contact with only 27 of these families despite clinic staff being required to attend work. There were no established criteria defining who should be contacted or how they should be evaluated during the crisis but rather clinicians made an intuitive decision as to who may require assistance. The response to the mailed questionnaire was poor (17/27). The eight families who received a follow up telephone call all returned the call but again there was no indication that any specific criteria were used in planning who should receive a telephone follow up in addition to the mailed questionnaire. This illustrates a haphazard approach during the time of clinic closure.

The loss to follow up rate was much greater over the SARS period compared to the non-SARS period 2 years later. It appears from this study that to maintain patient engagement in a clinic, there is a requirement not only for a mail out but also a telephone call. As this is a retrospective study we were not able to ascertain the reasons for not returning to the clinic however some parents expressed lingering concerns about the risk of exposure to an infectious disease in an outpatient clinic.

Although our results suggest that making a new neurological diagnosis may require a face to face assessment of the patient our study was not designed to look at this question and further research is required to determine if this is in fact true. If so, such an assessment may be facilitated through a telehealth assessment as opposed to a telephone interview.

The validity of using the parent completed ASQ system as an alternative approach to evaluate developmental outcome has been extensively evaluated [19-22] and has the advantage of being a cost-effective and simple screening tool for long term follow up of preterm high risk infants [23]. Some literature has suggested that parents may be unable to complete questionnaires satisfactorily due to reading, organizational, mental health and cognitive disabilities [19]. A limitation of this study is that we did not obtain socio-economic data of the parents as a determinant of the rate of ASQ reply and the accuracy of reporting developmental delay in their infants. It has been previously shown [24] however that the accuracy of parent reporting is not influenced by socio-demographic factors or maternal educational level. A further limitation is the potential difference in populations between the SARS and non-SARS groups as they were recruited in different time periods which may have affected the accuracy of our result. Future studies may compare alternative assessment types at the same time period.

Our rate of detection of developmental delay may have been ameliorated by adding other screening tools during the telephone contact. Further research should be performed to test the accuracy of different assessment systems when the gold standard of face to face assessment cannot be provided.

This study underscores the need for advance planning as to how best to provide outpatient care or assessment to families during an infectious disease outbreak. If there is no a priori planning there is a risk of inconsistent patient management as was illustrated here. This applies not only to NNFU clinics but to all clinics providing care for patients with chronic health conditions. Well planned mail and telephone contact may be of assistance in providing patient care during the time of infectious disease containment measures particularly if staff is deployed to work in other areas. Such contact may also help with maintaining the engagement of families and return to clinic. Consideration should be given for remuneration of physician and other health care providers for this telephone assessment. If staffing was adequate a telehealth or internet based [25] assessment may be more appropriate to diagnose certain types of conditions. After the crisis has passed, workload adjustments may be required to enable rapid rescheduling of face to face assessments. These reflections are timely in view of the current focus of hospitals and public health authorities' planning for anticipated influenza pandemics. In conclusion, non-urgent care clinics should have a priori planning for identification, contacting and assessment of their high risk patients during the periods of clinic closure due to infectious diseases outbreaks.

## Competing interests

Authors declare that they have no significant competing financial, professional or personal interests that might have influenced the performance or presentation of the work described in this manuscript.

## Authors' contributions

NN participated in design of the study, collecting data, data interpretation, statistical analysis, and writing the manuscript. LW participated in data collection and writing manuscript. KO participated in data interpretation and writing manuscript. SU participated in study design, data interpretation, and writing the manuscript. All authors read and approved the final manuscript.

## Pre-publication history

The pre-publication history for this paper can be accessed here:

http://www.biomedcentral.com/1471-2431/10/51/prepub
